# Biodegradable Film Is Enriched with Pomegranate Seed Oil and Microalgae for Preservation of Cajarana (*Spondias dulcis*)

**DOI:** 10.3390/polym17030367

**Published:** 2025-01-29

**Authors:** Kalinny A. Alves, Railene H. C. R. Araújo, Adriano S. Silva, Evanilson S. Almeida, Ágda M. F. Oliveira, Nayara S. Rocha, Max C. Araújo, Thaisa A. S. Gusmão, José F. Lima, João M. P. Q. Delgado, Joseane F. Pereira, Romário S. Santos, Antonio G. B. Lima

**Affiliations:** 1 Academic Unit of Agricultural Sciences, Federal University of Campina Grande, Pombal 58840-000, Paraíba, Brazil; kalialves1607@gmail.com (K.A.A.); evanilsom46@gmail.com (E.S.A.); 2 Department of Food Engineering, Federal University of Campina Grande, Campina Grande 58429-900, Paraíba, Brazil; railene.herica@professor.ufcg.edu.br (R.H.C.R.A.); adriano.sant@professor.ufcg.edu.br (A.S.S.); nayara.santos@estudante.ufcg.edu.br (N.S.R.); max.cesar@ufcg.edu.br (M.C.A.); thaisa.abrantes@professor.ufcg.edu.br (T.A.S.G.); 3 Postgraduate Program in Plant Science, Rural Federal University of the Semiarid, Mossoró 59625-900, Rio Grande do Norte, Brazil; agdamalany@hotmail.com; 4 Center for Natural and Human Sciences, Federal University of ABC, Santo André 09210-580, São Paulo, Brazil; josefranciraldo@gmail.com; 5 Institute of R&D in Structures and Construction (CONSTRUCT-LFC), Department of Civil Engineering, Faculty of Engineering, University of Porto, 4200-465 Porto, Portugal; 6 Department of Mechanical Engineering, Federal University of Campina Grande, Campina Grande 58429-900, Paraíba, Brazil; joseaneuepb@gmail.com (J.F.P.); romariofisico@gmail.com (R.S.S.); antonio.gilson@ufcg.edu.br (A.G.B.L.)

**Keywords:** post-harvest conservation, *Chlorella* sp., *Scenedesmus obliquus*, *Spirulina platensis*, mechanical properties

## Abstract

This study aimed to develop and characterize biodegradable films made from pectin, pomegranate seed oil, and different microalgae (*Spirulina platensis*, *Chlorella* sp., and *Scenedesmus obliquus*) and to evaluate their applicability as packaging by verifying their effect on the conservation and postharvest quality of cajarana (*Spondias dulcis*). The films proposed in this study were assessed for their physical, optical, and mechanical attributes, as well as the physicochemical characteristics of the fruits coated with the films after 14 days of storage at 10 ± 1 °C and relative humidity of 60 ± 5%. Incorporating microalgae improved the homogeneity and mechanical properties, decreasing breaking stress, elastic modulus, and maximum tensile strength, contributing to a lower solubility and improving the barrier properties of the films compared to the control (T1). The film formulated with 6% citric pectin, 40% glycerin, 0.5 mL·L^−1^ pomegranate seed oil (PSO), and 0.05% *Scenedesmus obliquus* showed better performance in solubility, water vapor permeability (WVP), and mechanical properties, maintaining gloss and transparency, approaching the performance of the commercial PVC film. The film was formulated with 6% pectin + 40% glycerin + 0.5 mL·L^−1^ PSO + 0.05% *Chlorella* sp. maintained the postharvest quality of cajarana fruits, allowing the conservation of the physicochemical quality of the fruits after 14 days of storage at 10 ± 1 °C and 60 ± 5% RH.

## 1. Introduction

A global shift is underway towards sustainable packaging solutions, emphasizing biodegradable alternatives to conventional petroleum-based plastics. This shift aims to mitigate post-harvest losses, and the escalating environmental pollution associated with traditional plastics [1,2]. Biopolymers offer a promising avenue for developing films and coatings, serving as effective carriers for various functional ingredients. These include antimicrobials, antioxidants, dyes, nutraceuticals, minerals, vitamins, pigments, and flavorings. By incorporating biopolymers into film formulations, it becomes possible to enhance functionality, improve material properties [3], inhibit food spoilage, extend shelf life, and significantly contribute to food preservation [4].

This study added the films with pomegranate seed oil (PSO), microalgae, and pectin. Pectin is a group of plant-derived complex carbohydrates composed of multiple hydrogalacturonic acid units. Pectin is a biopolymer that is useful in the food industry because it is water-soluble and commonly used as a gelling agent, emulsifier, thickener, etc. Pectin can be applied directly to foods or used as an edible coating as a preformed film over food [5]. PSO is a functional and nutraceutical oil with extensive bioactive capacity, including cytotoxic effects, antioxidant activity, and antimicrobial ability [6]. Its potential efficacy is linked to its distinct composition of fatty acids, notably rich in conjugated linolenic acid isomers, with a particular emphasis on punicic acid, and the presence in its composition of other essential antioxidant compounds, such as polyphenols, gallic and ellagic acid, and phytosterols [7,8,9]. It has piqued interest due to its versatile applications as a functional ingredient, a fat substitute, or in the manufacture of food packaging [9,10]. The nutritional and medicinal properties were explored for applications in edible and biodegradable coatings, including enhancing their emulsification capabilities and enriching fruits with beneficial health attributes [9,11,12,13,14].

Microalgae, in turn, are prized natural resources because of their wealth in nutrient content and bioactive compounds. They are rich in proteins, amino acids, peptides, polysaccharides, essential fatty acids, carotenoids, and phycocyanins, all of which possess functional properties [15]. Among the properties developed by microalgae, we can mention their wide applications and antioxidant, antimicrobial, and anticancer effects [16]. Notably, microalgae are a significant source of structural biopolymers, such as proteins and carbohydrates, which may exhibit a range of technological functionalities. They show potential as texturizers, stabilizers, or emulsifiers [17].

Recent research has explored extracts derived from *Scenedesmus* sp., *Spirulina platensis*, and *Chlorella* sp. in developing innovative coatings for postharvest fruit preservation. Formulations tested have demonstrated significant potential in maintaining fruit quality by enhancing the incorporation of bioactive compounds, mitigating the risk of pathogenic microorganism contamination, conferring functional properties to the coating, and improving the film’s physical and mechanical properties [18,19,20].

Cajarana (*Spondias dulcis*) is an ellipsoidal drupe, highly appreciated due to its excellent sensory characteristics, being rich in fiber, mineral salts (magnesium, potassium, zinc, copper, calcium, phosphorus, and iron), and some vitamins (A, B1, B6, and C) [21,22]. In addition, they have a high potential for the fresh fruit market due to their sensory and nutritional characteristics. However, most of it is intended for processing in the agroindustry with different applications (juices, cocktails, smoothies, liqueurs, and ice cream) [23,24].

Despite its attractive characteristics for consumption and processing, it has characteristics that mainly limit its exploitation in nature: it is highly perishable, has a short marketing period, and presents high rates of post-harvest losses [25]. These limitations can be mitigated through the application of technological measures, such as the use of coating [9,26], film for use as packaging [26,27], and gaseous preservation technology [28], among others. However, most preservation techniques have their disadvantages. Therefore, it is necessary to study and develop technologies so that fresh fruits can be marketed for a more extended period and preserve good sensory, nutritional, and biological quality [29].

Therefore, this study’s objective is to develop and feature biodegradable films enriched with pomegranate seed oil and different microalgae (*Spirulina platensis*, *Chlorella* sp., and *Scenedesmus obliquus*) and then evaluate their applicability as packaging, verifying their effect on the conservation and post-harvest quality of cajarana (*Spondias dulcis*).

## 2. Materials and Methods

### 2.1. Materials

Pomegranate seed oil provided by Oliveira et al. [8] was extracted using anhydrous and hydrated ethanol (90 °GL) in a Soxhlet extractor. Citric Pectin ATM (Adicel^®^, Belo Horizonte, Brazil) and glycerin (ACS científica) were purchased from local stores and microalgae (*Spirulina platensis*, *Chlorella* sp., and *Scenedesmus obliquus*) were provided by the company J.H. de Lima, CNPJ 23.176.796/0001-33 and processed in the form of powdered microparticles [30] in reduced-size biomass [20]. Cajarana (*Spondias dulcis*) fruits were purchased and collected from a domestic orchard in Pombal, Paraíba, Brazil.

### 2.2. Preparation of Film Solutions

All the film solutions evaluated herein (with some modifications) were prepared according to Espitia et al. [31]. A 200 mL amount of distilled water was heated to 80 ± 2 °C, and the pectin (6%) was diluted in the heated water with constant stirring until completely homogenized. After cooling to 35 °C, glycerin (40%), PSO (0.5 mL·L^−1^), and microalgae (0.05%) (*Spirulina platensis*, *Chlorella* sp. and *Scenedesmus obliquus*) were sequentially added. After adding all the raw materials, stirring was performed for 5 min for complete homogenization. Subsequently, the film-forming solutions were placed on glass plates measuring 39.7 cm^2^ and remained at the environment temperature (25 °C and 50 ± 3% RH) to complete drying.

### 2.3. Experimental Design

An experimental design was completely randomized to characterize the films with four treatments (T) and four replicates. The following formulations were tested: T1: 6% pectin + 40% glycerin + 0.5 mL·L^−1^ PSO; T2: 6% pectin + 40% glycerin + 0.5 mL·L^−1^ PSO + 0.05% *Spirulina platensis*; T3: 6% pectin + 40% glycerin + 0.5 mL·L^−1^ PSO + 0.05% *Chlorella* sp.; T4: 6% pectin + 40% glycerin + 0.5 mL·L^−1^ PSO + 0.05% *Scenedesmus obliquus*.

### 2.4. Characterization of Films

#### 2.4.1. Scanning Electron Microscopy (SEM)

The study utilized a scanning electron microscope to investigate the morphology of the films, examining both surface features and cross-sectional analysis (Tescan model VEGA 3 LMU, Brno–Kohoutovice, Czech Republic) operating at 0.3 kV and 300× magnification. The samples were fractured in liquid nitrogen to avoid plastic deformation during cross-section analysis.

#### 2.4.2. Thickness

Film thickness was determined using an external manual micrometer (110.284-NEW, Digimess, São Paulo, Brazil) with a precision of 0.01 mm. Measurements were taken at four locations: the film’s center and three equidistant points near the edge.

#### 2.4.3. Solubility

Film solubility was determined as described by Ge et al. [32]. The samples were prepared in quadruplicates, cut with 2 × 2 cm dimensions, and weighed. Before solubility testing, films were dried in an air circulation oven at 105 °C for 24 h to obtain their initial dry mass. Subsequently, the dried samples were immersed in 30 mL of distilled water at 25 °C for 24 h. The samples were dried again at 105 °C for 24 h. The percentage of mass loss was calculated based on the initial and final dry masses.

#### 2.4.4. Films Color Parameters

The films’ color analysis was performed using a colorimeter (Konica Minolta model Chroma meter CR-400, Ramsey, NJ, USA) configured in the L*, a*, and b* systems. The readings were taken on white sulfite paper. From the L*, a*, and b* values, the hue angle (°h) (Equation (1)) and the chroma saturation index (C*) (Equation (2)) were calculated.

(1) $${}^{\circ}h=arctan\left(\frac{b^{*}}{a^{*}}\right),$$

(2) $$C^{*}=\sqrt{a^{2}+b^{2}},$$

#### 2.4.5. Water Vapor Permeability (WVP)

Water vapor transmission rate (WVTR) was measured using the gravimetric method as outlined in ASTM E96/E96M–16 [33] and adaptations of the methodologies of Souza et al. [34]. The films were affixed to circular glass capsules with a diameter of 4 cm, inside which dry silica was placed at 0% relative humidity. The capsules were then transferred to desiccators maintained at 25 °C. These desiccators were filled with a saturated NaCl solution, establishing a relative humidity of approximately 75% outside the capsules. Samples were weighed at 24 h intervals for a maximum of seven days or until a 4% weight gain of the silica was observed. The analysis was performed in quadruplicate, and a test specimen (blank) prepared with silica was added, which allowed the variation in the weight of the material to be discounted or added. The water vapor permeability was calculated according to Equation (3):

(3) $$WVP=\frac{AW\times FT}{S\times \Delta p\times DUR},$$

where AW: film weight loss per hour (g/h); FT: film thickness (mm); S: area of exposed film (m^2^); Δp: tabled water vapor pressure (kph) at 25 °C; DUR: difference in relative humidity (initial relative humidity minus the average relative humidity over the seven days).

#### 2.4.6. Mechanical Tests

The films were subjected to quadruplicate tensile strength and perforation tests using a Texture Analyser TA.XT.plus texturometer (Stable Micro Systems Ltd., Vienna Court, UK). Samples were cut to 6.5 cm long and 3 cm wide for the tensile strength test, following the ASTM D-882-12 standard method [35]. The Texture Expert program calibrated the device for a test return distance of 20 mm. The samples were pulled at a speed of 0.21 mm·s^−1^, using a load cell with a capacity of 500 N in the equipment. The maximum stress, rupture stress, ductility, toughness, and modulus of elasticity were analyzed. For the perforation test, film dimensions were standardized to 8 × 8 (cm), following the standard ASTM F 1306-90 method [36]; the samples were fixed to the probe and perforated with a rounded tip probe moving at 0.21 mm·s^−1^ until the films were perforated, to obtain the maximum force at film rupture.

### 2.5. Treatments and Application of Films on Cajarana Fruits (Spondias dulcis)

The fruits (*Spondias dulcis*) were obtained from a domestic orchard in Pombal, Paraíba, Brazil. Fruits were harvested in the late afternoon. Only physiologically mature fruits were selected, excluding those with disease symptoms, pathogen presence, or any signs of mechanical damage. The selected fruits were packed in plastic boxes in a single layer and transported to the Post-harvest Laboratory at the Federal University of Campina Grande, Pombal campus. Upon arrival, they were subjected to a second selection process to ensure uniformity in size and color, and any fruit with defects or visible injuries was discarded. The fruits were then washed with a 1% neutral detergent solution. Following rinsing, they were sanitized by immersion in a sodium hypochlorite solution containing 100 ppm of chlorine for 15 min. After a final rinse with running water, the fruits were air-dried on sanitized benches.

This experiment was performed to test the applicability of the films in the conservation of cajarana fruits, and a completely randomized experimental design was implemented, composed of six treatments (T) and five replicates (16 fruits per experimental plot). The treatments applied were: T1: 6% pectin + 40% glycerin + 0.5 mL·L^−1^ PSO; T2: 6% pectin + 40% glycerin + 0.5 mL·L^−1^ PSO + 0.05% *Spirulina platensis*; T3: 6% pectin + 40% glycerin + 0.5 mL·L^−1^ PSO + 0.05% *Chlorella* sp.; T4: 6% pectin + 40% glycerin + 0.5 mL·L^−1^ PSO + 0.05% *Scenedesmus obliquus*; T5: polyvinyl chloride (PVC) film and T6: control (fruits that were not coated). The fruits were placed on polystyrene trays to which the treatments (T) were applied. They were then stored in a BOD incubator (Eletrolab, São Paulo, Brazil) for 14 days at 10 ± 1 °C and RH of 60 ± 5%. After storage time, physical–chemical analyses were performed. For this, the fruits were manually pulped and processed in a domestic centrifuge (Mondial, Turbo Juicer, Barueri, Brazil).

#### 2.5.1. External Appearance

External appearance was assessed using a 5-point scale: (4 = fruit free of stains and with a fresh appearance, 3 = signs of initial wilting (loss of shine and turgor) and stains on up to 5% of the surface, 2 = stains on 6% to 20% of the surface and initial wrinkling, 1 = stains on 21% to 40% of the fruit and advanced wrinkling (moderate intensity) and 0 = stains on more than 40% of the fruit and wrinkling at severe intensity and rot), with five untrained evaluators.

#### 2.5.2. Loss of Fresh Mass

A semi-analytical electronic scale (Bioprecisa^®^, Curitiba, Brazil) was employed to measure the weight of each fruit at the start and end of the storage period. Weight loss was expressed as a percentage, calculated by comparing the initial weight of the fruit to its weight at the end of storage.

#### 2.5.3. Shell Coloration

The color (L*, a*, and b*) of the fruit skin was determined as previously described (Section 2.4.4). Color difference (ΔE) (Equation (4)) was determined by the color difference (L*, a*, and b*) between the values recorded for the fruits after storage (to) and those obtained for the fruits at harvest (ti). Through the values DL* (Equation (5)), Da* (Equation (6)), and Db* (Equation (7)), it is possible to know the tendency of the color change in the fruits about the color at harvest.

(4) $$\Delta E={\left({\left({DL}^{*}\right)}^{2}{+\left({Da}^{*}\right)}^{2}+{\left({Db}^{*}\right)}^{2}\right)}^{\frac{1}{2}},$$

(5) $${DL}^{*}={L^{*}}_{ti}-{L^{*}}_{to},$$

(6) $${Da}^{*}={a^{*}}_{ti}-{a^{*}}_{to},$$

(7) $${Db}^{*}={b^{*}}_{ti}-{b^{*}}_{to},$$

#### 2.5.4. Soluble Solids, Titratable Acidity, SS/TA Ratio, and pH

A digital refractometer (Highmed, São Paulo, Brazil) was used to directly measure the soluble solids content in the cajarana fruit pulp, following the protocol described in [36], 3 mL of pulp was titrated with 0.1 M sodium hydroxide (NaOH) solution under constant stirring to determine titratable acidity. The results were expressed as a percentage of citric acid, as detailed in [37]. The soluble solids/titratable acidity (SS/TA) ratio was calculated. The pH of the homogenized pulp was measured directly using a benchtop digital pH meter (Digimed DM-22, São Paulo, Brazil) [36].

#### 2.5.5. Vitamin C

To determine vitamin C content, 5 mL of fruit pulp was diluted with 45 mL of oxalic acid. This diluted sample was titrated with a 2,6-dichlorophenolindophenol (DFI) solution under constant stirring. Following Tillman’s method, the results were expressed in percentage of ascorbic acid [37].

#### 2.5.6. Total Sugars

The total sugar content was determined by adopting the anthrone method described by Yemm and Willis [38] determined the total sugar content with modifications. Approximately 0.1 g of pulp was diluted in 50 mL of distilled water, using a 0.1 mL aliquot, adding 2 mL of anthrone under stirring, and placed in a bath at 100 °C for 5 min. After cooling, the absorbance of the samples was determined in a spectrophotometer (Thermoscientific/Genesys 20, Waltham, MA, USA) at a wavelength of 620 nm. The results were expressed in g·100 g^−1^.

### 2.6. Statistical Analysis

Analysis of variance (ANOVA) was conducted on the data. When statistically significant differences were observed, the Tukey test (*p* ≤ 0.05) was applied for multiple comparisons. All statistical analyses, including ANOVA and Tukey’s test, were performed using SISVAR version 5.6 [39]. Principal component analysis was also conducted using the statistical software R, Version 4.2.2 [40].

## 3. Results and Discussion

### 3.1. Visual Appearance and Morphological Characteristics

**Figure 1:**
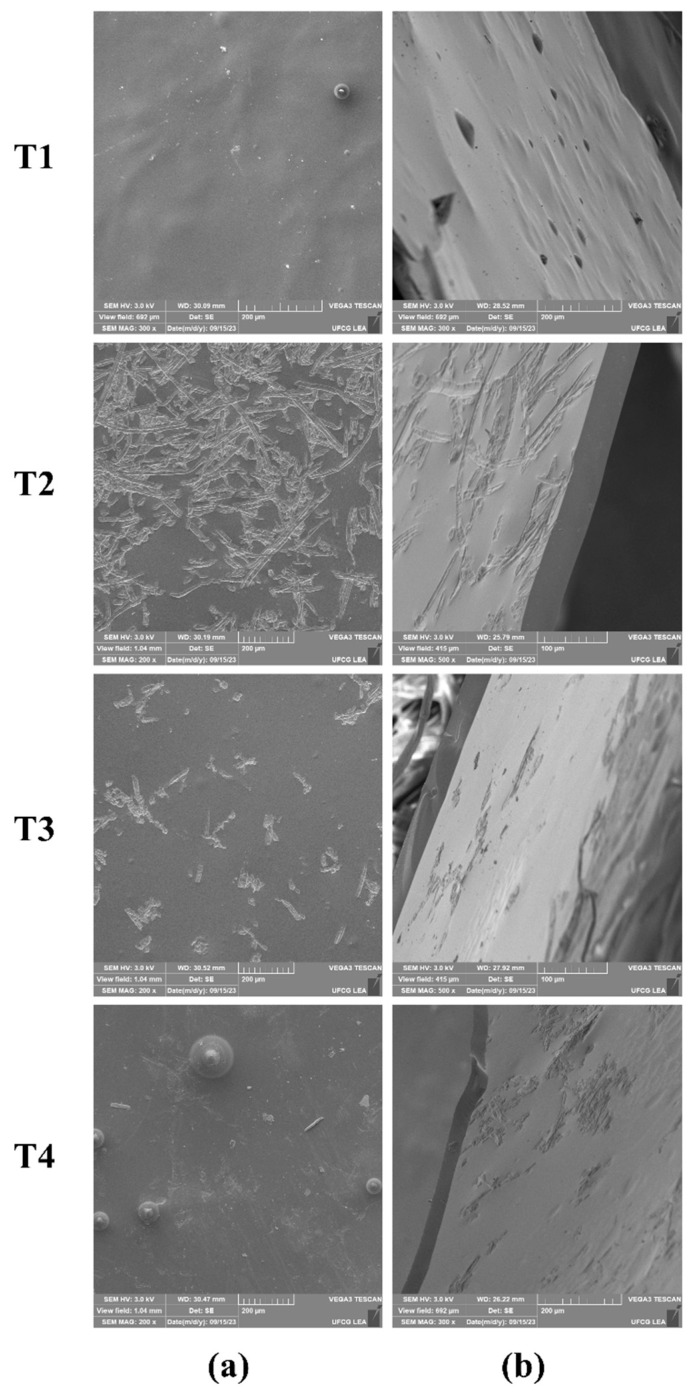
Micrographs of the surface (**a**) and cross-section (**b**) of films produced with pomegranate seed oil and microalgae (*Spirulina platensis*, *Chlorella* sp., and *Scenedesmus obliquus*) to be stored for 14 days at 10 ± 1 °C and 60 ± 5% RH. T1: film added with 0.5 mL·L^−1^ PSO. T2: film added with 0.5 mL·L^−1^ PSO + 0.05% *Spirulina platensis*; T3: film added with 0.5 mL·L^−1^ PSO + 0.05% *Chlorella* sp.; T4: film added with 0.5 mL·L^−1^ PSO + 0.05% *Scenedesmus obliquus*.

Without adding microalgae, the film surface displayed a consistent nanometric structure, resulting in a uniform, homogeneous, and crack-free appearance. These films were colorless and translucent, with a visually appealing appearance. None of the films presented insoluble particles visible to the naked eye, indicating purity and uniformity. In terms of handling and continuity, all films were similar, reinforcing the consistency of the results. Structurally, the films demonstrated a compact and homogeneous composition without visible pores or cracks (Figure 1a), indicating structural integrity.

The incorporation of microalgae into the films significantly enhanced their properties. Scanning electron microscopy revealed distinct surface morphologies: films containing *Chlorella* sp. exhibited an equidistant and asymmetric microstructure, implying that the biomass’s cellular components may interact (Figure 1a, T3). The presence of *S. obliquus* resulted in films with a heterogeneous distribution of structural elements characterized by the superposition of multiple layers in different regions. These unique structural features, likely influenced by the distinctive cellular components of *S. obliquus*, contributed significantly to the enhanced film resistance (Figure 1a, T4) [20].

The cross-section images (Figure 1b) confirm these observations, highlighting the importance of microalgae’s incorporation in the films’ structure, contributing significantly to their homogeneity. Obtaining more homogeneous surfaces may be related to more excellent compatibility between the raw materials used in the film formulations, indicating better interaction and resulting in better mechanical properties. Thus, the uniform internal structure of the films added with microalgae, especially T3 and T4, suggests that pectin does not tend to segregate in its presence, proving to be favorable to the interaction between the components of these formulations [41].

### 3.2. Colorimetric Parameters of the Films

The color parameters of packaging films are an essential property when it comes to consumer acceptance. Luminosity indicates the degree of clarity or brightness; the biodegradable films developed in this study showed high luminosity. A variation of 90.10 (T2) to 90.71 (T4) of the samples for this parameter was observed (Table 1). Characterized as a film with high brightness and transparency, it can indicate an excellent visual presentation. It is visually similar to commonly used plastic films, such as PVC (L* ≅ 99.00), an essential parameter for consumer acceptance since the material’s transparency allows one to visualize the packaged product [42].

The biodegradable films proposed in this study presented lower color saturation, with values ranging from 1.66 to 2.94, in agreement with the luminosity result, conforming to the transparency of these materials since neutral colors have low saturation (Table 1). The chroma values obtained in this study were lower than those reported by Silva et al. [43] for films made from ripe banana peel and starch enriched with *Eriobotrya japonica* leaf extract 13.2 to 28. While this indicates less intense coloration, it may not be a limitation, as it could potentially reduce photooxidation of the packaged food.

The hue angle (°h) varied from 122.20 to 129.52 and showed no difference between treatments (Table 1). The data obtained in this study exhibited a degree of similarity to those reported by Silva et al. [43], in which the films presented higher values for °h and lower values for C*, which is considered a positive indication regarding the film’s transparency.

Consumer acceptance of biodegradable films for food packaging is strongly influenced by their optical properties, including color, opacity, and light transmittance. Consumers generally prefer transparent and light-colored films. These optical properties not only enhance the visual appeal of the packaged food but also protect it from light damage. In the case of fruit packaging, the ability of biodegradable films to block UV light can effectively slow down the ripening process [44].

### 3.3. Thickness, Water Vapor Permeability and Solubility

Thickness control is difficult, especially in casting-type production processes, when working with viscous film-forming solutions, as it makes spreading over the plate difficult. However, the casting technique used to obtain the films in this study was efficient, resulting in uniform thickness, as demonstrated by the thickness values presented in (Table 1), receiving an average value of (0.06 mm) for all the films tested. The thickness of the film is a property indicative of the material’s uniformity and the consistent distribution of constituents throughout the film. Still, it is also an essential characteristic of the material intended for packaging because it significantly affects its mechanical performance, barrier and permeability properties, and optical properties [45,46].

Thickness is a parameter that depends on several factors, and because of this, a wide range of variation is found in the literature for biodegradable films [41,47,48,49,50,51]. The results determined in this investigation are comparable to the value reported in the study by Hiremani et al. [52], who, when developing bioactive and biodegradable chitosan/white saffron films, found thickness values ranging from 0.05 to 0.06. Schaeffer [53] assessed the characteristics of biodegradable films formulated from cassava starch and cornstarch, comparing these findings with those of biodegradable films already available. The commercial biofilm obtained a thickness of 0.03 mm, superior to the biofilms developed with thickness ranging from 0.10 to 0.13 mm.

The permeability to water vapor of the films produced in this research ranged from 0.778 to 0.782 (g·mm/m^2^·h·kPa), statistically equal (Table 1). Developing packaging materials with sufficient oxygen permeability properties is crucial to limit nutrient oxidation and maintain the quality of food products [54]. Salazar et al. [55], while examining the water vapor permeability of pectin films derived from fruits embedded with silicon dioxide nanoparticles, presented maximum values of 3.19 ± 2.61 g·mm/m^2^·h·kPa (orange pectin films) and 2.69 ± 0.08 g·mm/m^2^·h·kPa (mango pectin films), demonstrating that the films proposed in this study offer a more efficient barrier to water vapor.

The ability of biodegradable films to control water vapor transfer is critical for their effectiveness as food packaging. Low water vapor permeability is typically essential to minimize moisture uptake by the food and extend shelf life [56]. However, higher permeability is necessary for certain types of fresh produce to prevent water vapor condensation within the package, which can lead to microbial growth and subsequent food spoilage [50]. This highlights the importance of balancing permeability requirements based on the specific needs of the packaged food [57].

The solubility results of the biodegradable films developed in this study were consistent with the polymeric matrix’s nature, with high solubility, presenting values ranging from 63.17 to 92.88% (Table 1). Similar values were obtained by González-Sandoval et al. [58], who found solubility values ranging from 81.3% to 91.0% for films with *Opuntia* spp. mucilages, and Lin et al. [59] reported a wide range of water solubility values for films incorporating pectin and chitosan, from 19.63% to 86.30%. This high solubility and its variations elucidate the different materials used in the polymer matrix in film formulations [60]. It is also worth noting that pectin has high hydrophilicity due to the distribution of hydrophilic and hydrophobic groups in its structure and the low level of crosslinking of the polymer, which facilitates the penetration of the solvent into the polymer phase [59].

Evaluating moisture content, water vapor permeability (WVP), and solubility is crucial for assessing the suitability of films for food packaging applications. These properties should be minimized to limit moisture exchange between the food and the environment while preserving food quality [59]. Numerous factors influence these characteristics, including polymer properties, film matrix integrity, interactions within the film formulation (for example, polymer–plasticizer interactions), and processing conditions. WVP is intricately linked to solubility, moisture content, and film thickness [61].

### 3.4. Mechanical Tests

The values obtained for the rupture tension demonstrate that the biodegradable films (T1, T2, T3, and T4) were relatively higher than the data obtained by the commercial PVC film (T5), confirming the more excellent resistance of the developed materials. Among them, T1 (0.068) and T2 (0.062) stand and are statistically equal (Table 2). Determining mechanical properties is essential in the characterization of films, as it can indicate the stability, functionality, and applicability of the developed materials [62] and their ability to guarantee integrity during the transportation, control, and storage of packaged foods.

Rupture tension (ductility) varied from 9.77 to 19.23%, and there was a statistical difference between the treatments. The commercial PVC film (T5) stood out over the other biodegradable films proposed in this study (T1, T2, T3, and T4), showing superiority to elasticity (Table 2). Films produced from pectin typically exhibit limited extensibility due to the linear arrangement of pectin molecules within the solid state and in solution. While our research did not observe increased rupture strength by incorporating microalgae biomass, previous studies have demonstrated contrasting results. Carissimi et al. [61] reported that adding microalgae to starch-based films decreased rigidity and resistance while enhancing flexibility. This observation aligns with the plasticizing effect attributed to microalgae, further supported by an increase in film hydrophilicity, a common characteristic of plasticizers.

The treatments studied significantly influenced the tenacity. The values obtained by the biodegradable films T1 and T2 were relatively higher than those of the other treatments (Table 2). This result confirms the results obtained in the breaking tension parameter, reducing the superiority of these films in terms of resistance: their capacity to absorb energy and deform without fracturing.

The modulus of elasticity, also known as Young’s modulus, was significantly influenced by the treatments and ranged from 0.35 to 1.10 MPa (Table 2). The PVC treatment (T5) presented the lowest value for this parameter, indicating that it is more elastic than the others. Among the biodegradable films proposed in this study, it found that the control treatment (T1) without the addition of microalgae presented the highest value (1.10 MPa), statistically equal to T2 with (0.90 MPa), indicating that they are more rigid than the others. However, it is more flexible than the film developed by Gonçalves et al. [63], who, when studying the structure and functional properties of films created with varying concentrations of cellulose acetate and glycerol, found values for Young’s modulus that ranged from 5 to 6.62 MPa.

The proposed treatments significantly influenced the maximum tensile strength. The biodegradable films proposed in this study (T1, T2, T3, and T4) were more resistant than PVC (T5) due to the lower force (N) required for their rupture. T1 and T2 presented the highest values and were statistically equal (Table 2). Typically, pectin-based biofilms present high tensile strength and slight elongation since the chains of this polysaccharide are essentially linear, frequently described in the literature as brittle and requiring plasticizers to enhance their flexibility [5,64].

### 3.5. Application of Films on Cajarana Fruits (Spondias dulcis)

Cajaranas (*Spondias dulcis*) were initially characterized at the experiment installation (*n* = 10). The values of luminosity (L*) were obtained in the harvested fruits at 52.38. The chromaticity index (C*) presented values of 34.14. The hue color angle (°h) presented values of 114.90, with an external appearance of 4.0. As well as the other values of the physical–chemical parameters can be observed in (Table 3). According to Jayarathna et al. [65], it was found that the cajarana fruits were harvested at the semi-ripe stage (II), with a break in the green color and considered excellent commercial quality.

**Figure 2:**
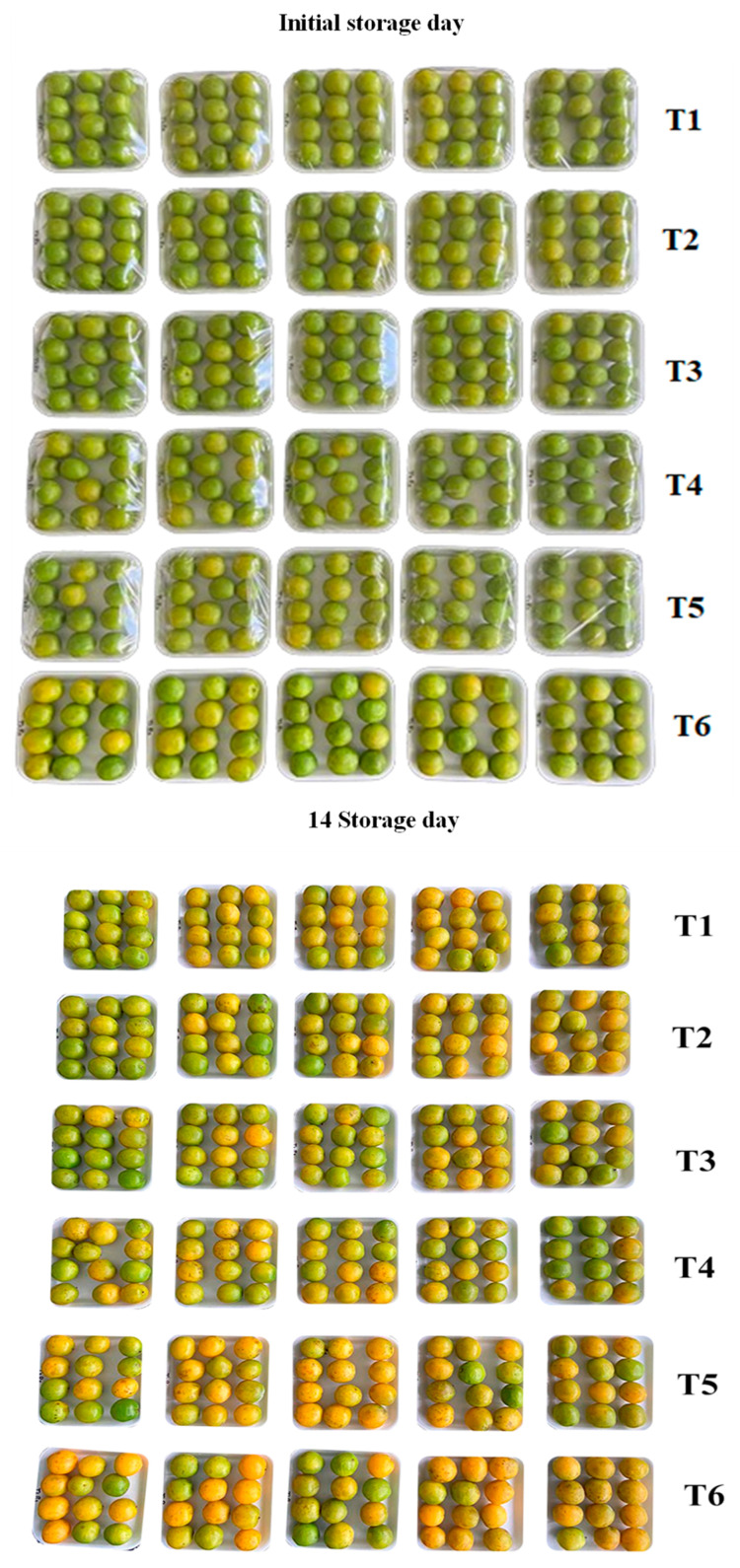
Comparison of cajarana fruits at the beginning of storage and after 14 days at 10 ± 1 °C and 60 ± 5% RH. T1: film added with 0.5 mL·L^−1^ PSO. T2: film added with 0.5 mL·L^−1^ PSO + 0.05% *Spirulina platensis*; T3: film added with 0.5 mL·L^−1^ PSO + 0.05% *Chlorella* sp.; T4: film added with 0.5 mL·L^−1^ PSO + 0.05% *Scenedesmus obliquus*. T5: PVC film; T6: control.

The biodegradable films were applied to the trays containing the cajarana fruits. After 14 days of storage at 10 ± 1 °C and 60 ± 5% RH, the fruits were analyzed, and Figure 2 shows the visual difference from the first day of storage and after 14 days.

The hue angle (°h) of the cajarana fruits was significantly influenced by the films tested after storage. It is possible to verify that the fruits coated with PVC (T5) and the control (T6) obtained the lowest averages for this parameter, 97.85 and 95.86, respectively. Additionally, it should be noted that the fruits exhibited a more pronounced yellow hue compared to the others (Figure 2 and Table 4). It is reported that after 14 days of storage, T4 provided the highest values and did not differ from T2 and T3, showing the potential of these films to delay the ripening of these fruits, proving to be more efficient by maintaining the color closer to green for a more extended period (Figure 2 and Table 4).

The alteration in the coloration of the fruit peel is ascribed to elevated ethylene production, which facilitates the upregulation of chlorophyllase enzyme activity [8]. This enzymatic activity is pivotal in the degradation of chlorophyll, which leads to a decrease in the characteristic green coloration and the pronounced yellowing of the fruits [66]. The application of biodegradable films (T2, T3, and T4) may have hindered the action of oxygen and increased the concentration of CO_2_ internally, resulting in the persistence of the green pigment to the detriment of PVC [67].

The luminosity (L*) and chromaticity (C*) of cajarana fruits after storage were significantly influenced by the films studied and showed similarities. The fruits coated with PVC (T5) and the control (T6) presented the highest values of L* (52.35 and 52.43), respectively, as well as for C* (32.05 and 32.67), and were statistically equal (Table 4). This can be explained in agreement with the °h; the fruits with these treatments were more yellowish and brighter than the others, indicating they were in a faster maturation process [68]. Control fruits (T6) considerably presented accelerated ripening during storage, promoting increased shine (Figure 2). Conversely, covered fruits (T2, T3, and T4) displayed more significant opacity, consequently slowing down the degradation of the pigments [69].

The fruits coated with biodegradable films (T1, T2, T3, and T4) presented the lowest values for color difference (CD) (Table 4); that is, at the end of the storage period, these fruits had a color similar to the fruits on the occasion of the harvest (Figure 2) and (Table 3). The treatments T5 (PVC) and T6 (control) presented the highest values for this parameter (Table 4); that is, the fruits with these treatments presented a more significant color difference compared to the fruits at the time of harvest (Table 3). Possibly, the proposed films reduced the natural processes of chlorophyll degradation that gave the green color to the fruits and inhibited the synthesis of carotenoid pigments. These yellow pigments characterize ripening [68,70], causing this color difference to be more minor in these treatments.

**Figure 3:**
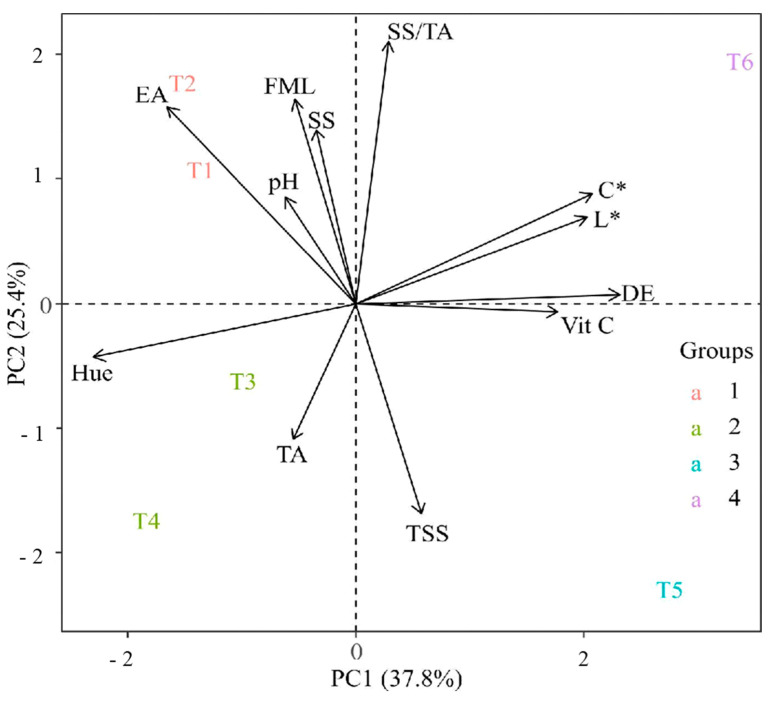
Principal component analysis (PCA) of cajarana fruits in biodegradable films produced with pomegranate seed oil and microalgae (*Spirulina platensis*, *Chlorella* sp., and *Scenedesmus obliquus*) after 14 days of storage at 10 ± 1 °C and 60 ± 5% RH.

In this study, external appearance (EA) scored 4.0. It was associated with an acceptable appearance for consumption, i.e., fruit free of blemishes and fresh appearance, obtained by untrained evaluators (Table 3). It can be inferred that the fruits packaged and covered with the proposed films presented a more acceptable appearance for consumption after 14 days of storage at 10 ± 1 °C and 60 ± 5% RH (Figure 3 and Table 4). Among the films studied, T1 stood out from the others, with an average of 3.40, i.e., the fruits of this treatment presented the best quality characteristics to appearance according to the evaluators, not differing statistically from T2 and T3 (Figure 3 and Table 4) (Figure 2 and Table 4).

The pH values of cajarana fruits were not significantly influenced by the treatments applied after 14 days of storage at 10 ± 1 °C and 60 ± 5% RH. Small oscillations were observed between treatments from 2.34 to 2.41, characterizing them as acidic (Table 4). The treatments also significantly influenced treatable acidity, ranging from 1.40 to 1.66. Film T1 produced fruits with a higher value of 1.66 and did not differ statistically from treatments T3, T4, and T5, indicating that the fruits of these treatments were less ripe than the others (Table 4).

A significant variation in soluble solids content was observed among the treatments. After 14 days of storage at 10 ± 1 °C and 60 ± 5% relative humidity, all treatments exhibited increased soluble solids. The lowest value was obtained in fruits coated with PVC (T5, 12.74%), not differing statistically from treatments T2 and T3 (Table 4). The SS/TA ratio increased slightly after storage in all treatments, oscillating from 8.16 to 9.58 (Table 4). Total sugars (TS) of cajarana fruits were also significantly influenced by the treatments applied after 14 days of storage at 10 ± 1 °C and 60± 5% RH, ranging from 9.28 to 11.05, obtained by treatments T4 and T1, respectively (Table 4). This indicates the potential not only of PVC but also of films T2 and T3, which may have acted in maintaining fruit respiration and in the consumption of sugars as a substrate in the respiratory process [8] and thus contributed to the preservation of soluble solids in these treatments. Notably, applying barriers, such as films, creates a modified atmosphere that can reduce carbohydrate metabolism, delaying starch degradation [71].

It was observed that the fruits subjected to treatment T2 and control T6 exhibited lower titratable acidity content and higher SS/TA ratio values throughout the storage period in comparison to the fruits from other treatments (Table 4). The organic acid content is higher in the fruits at the beginning of ripening, but as ripening progresses, there is a reduction. Consequently, there is an increase in the SS/TA ratio of the fruits due to the use of organic acids in the synthesis of sugars and as a substrate for respiration. Acidity content and higher SS/TA ratio values over the days of storage compared to the fruits of the other treatments (Table 4). The organic acid content is higher in the fruits at the beginning of ripening, but as ripening progresses, there is a reduction. Consequently, there is an increase in the SS/TA ratio of the fruits due to the use of organic acids in the synthesis of sugars and as a substrate for respiration [72].

The control treatment (T6) (9.19%) had a more pronounced fresh mass loss than the other treatments. The lowest fresh mass loss was obtained using PVC film (T5) (2.67%). The other films had intermediate fresh mass loss (T1, T2, T3, and T4) and were statistically equal (Table 4). This behavior of biodegradable films on the mass loss of cajarana fruits occurs mainly due to the hydrophilic characteristics of the polysaccharide-based films that do not form impermeable barriers. Therefore, processes such as respiration, transpiration, and oxidation occur, and consequently, the weight loss of the fruit is not yet efficiently controlled [73]. The results obtained in this study are similar to those reported by Alves et al. [74] when evaluating the post-harvest quality of Umbuzeiro fruits (*Spondias tuberosa Arruda*) stored with and without modified atmosphere and at different temperatures, reporting a more significant mass loss for the control fruits and the lowest mass losses for the fruits coated with PVC.

The vitamin C content of cajarana fruits showed statistical differences depending on the treatments applied. In addition, it is noteworthy that there was a reduction in ascorbic acid levels after 14 days of storage at 10 ± 1 °C and 60 ± 5% RH (Table 4). The highest content was found in fruits subjected to T6 control (9.20), not differing statistically from T5 PVC (8.63). The other biodegradable films tested were statistically equal and lower than T5 and T6 (Table 4). This fact differs from those obtained by Teodosio et al. [9] in a study with coatings based on *Chlorella* sp. associated with PSO. A decline in ascorbic acid content was observed during the 12-day storage period (14 ± 2 °C, 85 ± 5% RH) of *Spondias tuberosa* fruits, a phenomenon typical of ripening fruits. Nonetheless, coatings formulated with *Chlorella* sp. effectively preserved ascorbic acid levels, while the control treatment exhibited the lowest ascorbic acid content.

### 3.6. Principal Component Analysis (PCA)

The principal component analysis (PCA) between the responses of the physical–chemical variables of the cajarana fruits and the films studied is presented in (Figure 3). Principal components 1 (PC1) and 2 (PC2) explained 63.2% of the total variance between the variables, being responsible for 37.8% and 25.4%, respectively (Figure 3).

External appearance (EA) and pH were more closely related to treatments T1 and T2. Fresh mass loss (FML), SS/TA ratio, chromaticity (C*), luminosity (L*), color difference (CD), and vitamin C (Vit C) were more closely related to group T6. Soluble solids content (SS) was associated with T5. Total soluble sugar content (TS) and hue angle (°h) were strongly related to treatments T4 and T3. In addition, a positive relationship was observed between FML and SS and between CD and Vit C. TS was negatively associated with EA, pH, FML, and SS (Figure 3).

From the treatments studied, it was observed that four distinct groups could be detected and classified and were identified with different colors: group 1 (pink), group 2 (green), group 3 (blue), and group 4 (purple). Treatments T1 and T2 were grouped in group 1. Treatments T3 and T4 were grouped in group 2, suggesting a similarity. Treatment T5 was alone in group 3, and T6 was isolated in group 4, indicating that these treatments have distinct characteristics (see Figure 3).

## 4. Conclusions

In this work, a new biofilm has been developed, evaluated, and applied to fruit preservation. The innovative aspect is related to the insertion of different microalgae (*Spirulina platensis*, *Chlorella* sp., and *Scenedesmus* sp.) as components of the pectin matrix, which forms the biofilm. From the results, it was verified that:

The incorporation of microalgae particles contributed to the better structure of the biofilms as smart packaging for the post-harvest conservation of fruits because they are rich in peptides, amino acids, and antioxidants, which contribute to the potential for post-harvest conservation of fruits during storage. Furthermore, microalgae improved the homogeneity and mechanical properties of the films, resulting in a reduction in the tensile strength, modulus of elasticity, and maximum tensile strength. This contributed to lower solubility and improved the barrier properties of the films compared to the control sample (6% pectin + 40% glycerin + 0.5 mL·L^−1^ pomegranate seed oil).

The film formulated with 6% pectin, 40% glycerin, 0.5 mL·L^−1^ PSO, and 0.05% *Scenedesmus obliquus* showed better solubility, water vapor permeability (WVP), and mechanical properties. It maintained a gloss and transparency, approaching the performance of the commercial PVC film.

The film formulated with 6% pectin + 40% glycerin + 0.5 mL·L^−1^ PSO + 0.05% *Chlorella* sp. maintained the post-harvest quality of cajarana fruits, allowing the conservation of the fruits’ physical–chemical quality after 14 days of storage at 10 ±1 °C and 60 ± 5% RH.

## Figures and Tables

**Table 1:** Chromatic coordinates, thickness, water vapor permeability, and solubility of films produced with PSO and microalgae (*Spirulina platensis*, *Chlorella* sp., and *Scenedesmus obliquus*).

Parameters	T1	T2	T3	T4	CV (%)
L*	90.46 ± 0.33 ab	90.10 ± 0.25 b	90.68 ± 0.18 ab	90.72 ± 0.10 a	0.25
C*	2.80 ± 0.30 a	1.66 ± 0.11 b	2.85 ± 0.12 a	2.94 ± 0.20 a	7.73
°h	129.52 ± 5.60 a	125.40 ± 2.56 a	122.63 ± 0.68 a	122.20 ± 0.57 a	2.49
Thickness (mm)	0.06 ± 0.00 a	0.06 ± 0.00 a	0.06 ± 0.00 a	0.06 ± 0.00 a	-
WVP (g·mm/m^2^·h·kPa)	0.779 ± 0.001 a	0.779 ± 0.001 a	0.782 ± 0.006 a	0.778 ± 0.002 a	0.42
Solubility (%)	92.88 ± 10.50 a	85.31 ± 13.14 ab	91.98 ± 3.78 a	63.17 ± 12.30 b	12.70

Legend: means ± standard deviation; CV: coefficient of variation; WVP: water vapor permeability; T1: film added with 0.5 mL·L^−1^ PSO. T2: film added with 0.5 L^−1^ PSO + 0.05% *Spirulina platensis*; T3: film added with 0.5 mL·L^−1^ PSO + 0.05% *Chlorella* sp.; T4: film added with 0.5 mL·L^−1^ PSO + 0.05% *Scenedesmus obliquus*. Results in the same row followed by different letters (a, b) are significantly different by Tukey’s test (*p* < 0.05).

**Table 2:** Mechanical properties: tensile strength (TS), ductility (D), tenacity (T), modulus of elasticity (ME), maximum tensile force (MTF) of films produced with PSO and microalgae (*Spirulina platensis*, *Chlorella* sp., and *Scenedesmus obliquus*).

Parameters	T1	T2	T3	T4	T5	CV (%)
TS (MPa)	0.068 ± 0.009 a	0.062 ± 0.007 a	0.042 ± 0.004 b	0.040 ± 0.004 b	0.0170 ± 0.010 c	16.72
D (%)	11.46 ± 0.80 b	12.45 ± 2.56 b	9.77 ± 1.10 b	9.99 ± 2.16 b	19.23 ± 3.06 a	16.84
T (MPa)	0.0059 ± 0.001 ab	0.0066 ± 0.002 a	0.0023 ± 0.0008 c	0.0025 ± 0.0012 c	0.0028 ± 0.0009 bc	38.07
ME (MPa)	1.10 ± 0.11 a	0.90 ± 0.18 ab	0.65 ± 0.13 bc	0.59 ± 0.07 cd	0.35 ± 0.10 d	17.46
MTF (N)	8.82 ± 1.13 a	8.05 ± 0.86 a	5.37 ± 0.98 b	5.22 ± 0.55 b	2.87 ± 0.22 c	13.48

Legend: means ± standard deviation; CV: coefficient of variation; T1: film added with 0.5 mL·L^−1^ PSO. T2: film added with 0.5 mL·L^−1^ PSO + 0.05% *Spirulina platensis*; T3: film added with 0.5 mL·L^−1^ PSO + 0.05% *Chlorella* sp.; T4: film added with 0.5 mL·L^−1^ PSO + 0.05% *Scenedesmus obliquus*. T5: PVC film. Results in the same row followed by different letters (a, b, c, d) are significantly different by Tukey’s test (*p* < 0.05).

**Table 3:** Physical and physicochemical characteristics of fresh cajaranas (*Spondias dulcis*) after harvest.

Parameters	Cajaranas (*Spondias dulcis*)
L*	52.38 ± 2.31
C*	34.14 ± 1.49
h°	114.90 ± 2.06
External appearance	4.00 ± 0.00
Weight (g)	23.88 ± 1.20
Longitudinal diameter (mm)	33.76 ± 1.11
Cross diameter (mm)	36.90 ± 1.04
Treatable acidity (TA) (% citric acid)	1.57 ± 0.21
pH	1.99 ± 0.11
Total soluble solids (SS) (°Brix)	12.73 ± 0.24
SS/TA ratio	8.10 ± 1.18
Ascorbic acid (mg·100 mL^−1^)	12.14 ± 1.38
Total sugars (g·100 g^−1^)	10.74 ± 1.24

Legend: means ± standard deviation.

**Table 4:** pH, Soluble Solids (SS), Titratable Acidity (TA), SS/TA, Total Sugars (TS), Loss of Fresh Mass (LFM), Vitamin C (AA), Chromatic coordinates (°h, C*,L*), Color difference (CD) and External Appearance (EA) of films produced with PSO and microalgae (*Spirulina platensis*, *Chlorella* sp., and *Scenedesmus obliquus*).

Parameters	T1	T2	T3	T4	T5	T6	CV (%)
pH	2.41 ± 0.08 a	2.36 ± 0.12 a	2.34 ± 0.09 a	2.34 ± 0.05 a	2.35 ± 0.04 a	2.34 ± 0.15 a	3.97
SS	14.34 ± 0.52 a	13.20 ± 0.37 cd	13.42 ± 0.52 bcd	13.58 ± 0.25 bc	12.74 ± 0.18 d	14.04 ± 0.13 ab	2.69
TA	1.66 ± 0.090 a	1.40 ± 0.073 c	1.53 ± 0.096 abc	1.60 ± 0.045 ab	1.56 ± 0.045 ab	1.47 ± 0.070 bc	4.73
SS/TA	8.65 ± 0.63 bc	9.44 ± 0.57 ab	8.76 ± 0.35 bc	8.51 ± 0.28 c	8.16 ± 0.24 c	9.58 ± 0.42 a	4.96
TS	10.10 ± 0.51 a	9.28 ± 0.33 ab	10.63 ± 0.55 ab	11.05 ± 1.24 b	10.67 ± 0.85 ab	10.59 ± 0.58 ab	7.0
LFM	7.24 ± 0.31 b	6.99 ± 0.20 b	7.22 ± 0.37 b	7.32 ± 0.42 b	2.67 ± 0.08 c	9.19 ± 0.97 a	7.14
AA	8.29 ± 0.35 b	8.11 ± 0.66 b	8.17 ± 0.36 b	8.67 ± 0.40 b	8.63 ± 0.51 ab	9.20 ± 0.39 a	5.39
°h	103.38 ± 2.53 bc	106.12 ± 3.71 ab	108.13 ± 1.96 ab	109.14 ± 1.69 a	97.85 ± 4.23 cd	95.87 ± 2.22 d	2.78
C*	31.40 ± 0.56 ab	31.07 ± 1.20 ab	31.14 ± 0.56 ab	29.65 ± 0.82 b	32.05 ± 0.37 a	32.67 ± 1.49 a	3.41
L*	51.62 ± 1.11 ab	51.22 ± 1.05 ab	51.19 ± 0.67 ab	49.82 ± 1.19 b	52.35 ± 1.18 a	52.43 ± 1.08 a	2.12
CD	7.20 ± 1.33 b	6.31 ± 1.29 b	5.05 ± 0.78 b	6.06 ± 0.85 b	11.06 ± 1.27 a	11.54 ± 1.10 a	14.28
EA	3.40 ± 0.28 a	3.28 ± 0.23 ab	2.68 ± 0.30 abc	2.80 ± 0.42 bc	2.20 ± 0.40 c	2.60 ± 0.45 bc	12.62

Legend: means ± standard deviation; CV: coefficient of variation; T1: film added with 0.5 mL·L^−1^ PSO. T2: film added with 0.5 mL·L^−1^ PSO + 0.05% *Spirulina platensis*; T3: film added with 0.5 mL·L^−1^ PSO + 0.05% *Chlorella* sp.; T4: film added with 0.5 mL·L^−1^ PSO + 0.05% *Scenedesmus obliquus*. T5: PVC film; T6: control. Results in the same line followed by different letters (a, b, c, d) are significantly different by Tukey’s test (*p* < 0.05).

## Data Availability

Data are contained within the article.
